# In Vitro Comparison of the Wettability of a Bioceramic Root Canal Sealer on Dentin With and Without Erbium-Doped Yttrium Aluminum Garnet (Er:YAG) Laser Irradiation

**DOI:** 10.7759/cureus.23715

**Published:** 2022-03-31

**Authors:** Poonam Joshi, Rajesh Shetty, Arun Banpurkar, Dr. Vini Mehta, Gargi Sarode, Pranjali Yedewar, Tanvi Sharma

**Affiliations:** 1 Conservative Dentistry and Endodontics, Dr. D.Y. (Dnyandeo Yashwantrao) Patil Dental College and Hospital, Pune, IND; 2 Department of Physics, Savitribai Phule Pune University, Pune, IND; 3 Public Health Dentistry, People's College Of Dental Sciences and Research Centre, Bhopal, IND; 4 Oral Pathology and Microbiology, Dr. D.Y. (Dnyandeo Yashwantrao) Patil Dental College and Hospital, Pune, IND; 5 Public Health, Stat Sense, Pune, IND

**Keywords:** frequency, contact angle, laser, root canal sealer, bioceramic

## Abstract

Introduction: To evaluate and compare the wettability of bioceramic root canal sealer (BioRoot™ RCS, Septodont, Saint-Maur-des-Fossés, France) on dentin with and without erbium-doped yttrium aluminum garnet (Er:YAG) laser irradiation using different frequencies and energies.

Methods: A hundred single-rooted tooth samples were divided into five groups of 20 samples each. Each group was treated with different methods before sealer application as follows: Group 1: Treated with 2 ml 17% ethylenediaminetetraacetic acid (EDTA) irrigant for one minute (control group); group 2: Irradiated with Er:YAG laser for one minute (8 Hz frequency and 200mJ energy); group 3: Irradiated with Er:YAG laser for one minute (8 Hz frequency and 400mJ energy); group 4: Irradiated with Er:YAG laser for one minute (16 Hz frequency and 200mJ energy); group 5: (n=20) samples irradiated with Er:YAG laser for one minute (16 Hz frequency and 400mJ energy). Bioceramic root canal sealer application was done onto the treated dentin specimen using a micropipette. The contact angle of the drop of the sealer with the dentin surface was measured after five minutes using a contact angle analyzer.

Results: There was no significant difference in wettability between Er:YAG laser using 8Hz frequency and 200mJ energy and Er:YAG laser using 8Hz frequency and 400mJ energy. However, there was a significant difference between the other two groups of Er:YAG laser using 16Hz frequency and 400mJ energy and Er: YAG laser using 16Hz frequency and 200mJ energy.

Conclusion: Contact angle was found to be lowest in the group that was irradiated with Er:YAG laser for one minute (16 Hz frequency and 400mJ energy) before the sealer application. The test findings demonstrated that the control group had the highest contact angle (low wettability) and it was statistically significant with all other groups.

## Introduction

The basic goal of root canal (RC) therapy is eradicating microbes and debris in RCs to prevent further progression of infections or to treat the existing ones. This can be achieved via thorough instrumentation aiding in cleaning and shaping canals, then obturation of RC space with stable and non-toxic materials [1-3]. During the RC obturation, an RC sealer is required for achieving a seal that is fluid-tight in an uneven RC system [4].

Endodontic bioceramic materials are either bioactive or bioinert depending on how they interact with living tissue around teeth [5]. Bioactive materials are categorized as degradable or nondegradable based on their stability. According to Grossman, biological and physical properties of bioceramic-based RC sealers are that they should be sticky enough to ensure effective adhesion to canal wall post setting, must be able to create a hermetic seal, must show radiopacity for visibility on intraoral periapical (IOPA) radiographs, the powder particles must be minute for mixing easily with liquid, must not cause shrinkage post setting, it should not cause discoloration of teeth, must be bacteriostatic or not promote the growth of microbes at least, must take a longer time to set, must be insoluble with tissue fluids, and periapical tissue must tolerate it well. If the RC filling needs to be removed, it should be soluble in common solvents [6].

Flow and wetting are key qualities of RC sealers during obturation for optimal binding between the RC walls and the main root filling material, resulting in the development of a fluid-tight and bacteria-proof seal [7,8]. Flow is a key quality permitting sealers to cover inaccessible areas including dentine abnormalities, accessory canals, voids, and isthmus between master and accessory cones [7]. Temperature, particle size, and time of mixing are all factors influencing a sealer's flow rate [9-11]. Instrumentation quality and irrigant type used in the disinfection of RCs play an essential role. In the course of biomechanical preparation of the RC system, various chemical treatments have varying impacts on the biomechanical qualities of the dentin layer. Researchers found that alternating watering of canals along with ethylenediaminetetraacetic acid (EDTA) and sodium hypochlorite (NaOCl) was a highly effective strategy for removing the smear layer [12,13]. Irrigating solutions can alter the wettability of RC dentine, altering bacteria adhesion [14] and its interaction with restorative materials [15]. Wettability is measured by the contact angle (CA) established by linking a liquid drop and the planar surface of a solid. A practical measure of a liquid material's wetting behavior on a solid surface is the CA [16]. Because it is inversely proportional to wettability, the smaller the CA, the higher is surface free energy and, hence, the better the adhesion [17]. Relative wetting ability demonstrated by the intraradicular dentin surfaces determines R sealer adherence [18].

In recent times, the employment of lasers in endodontics is gaining importance, mainly in smear layer removal. Extensive research demonstrated successful smear layer removal using erbium-doped yttrium aluminum garnet (Er:YAG) laser without charring, melting, and recrystallization of dentine compared to other types of lasers [19-21]. Takeda et al. also pointed out that Er:YAG laser promoted exposure of the dentin tubules with increased dentin permeability [21]. These observations would justify a tight marginal sealing of RC filling materials post this laser usage. Thus, the need arises for extensive research on the wettability of bioceramic RC sealers with laser irradiation using the Er:YAG laser. There are only a handful of studies in the literature investigating the efficacy of this laser at various frequencies, energies, or intervals. Hence, the current study aimed to evaluate and compare the wettability of bioceramic RC sealer (BioRoot™ RCS, Septodont, Saint-Maur-des-Fossés, France) on dentin with and without Er:YAG laser irradiation using different frequencies and energies.

## Materials and methods

A total of 50 single-rooted permanent canal teeth were taken. After decoronation and apical resection, they were split buccolingually into 100 dentin sections of 2mm thickness each.

Grouping of samples

Group 1: (n=20) samples treated with 2ml 17% EDTA irrigant for one minute before sealer application (control group); Group 2: (n=20) samples irradiated with Er:YAG laser for one minute, using 8Hz frequency and 200mJ energy before sealer application; Group 3: (n=20) samples irradiated with Er: YAG laser for one minute, using 8Hz frequency and 400mJ energy before sealer application; Group 4: (n=20) samples irradiated with Er:YAG laser for one minute, using 16Hz frequency and 200mJ energy before sealer application; Group 5: (n=20) samples irradiated with Er:YAG laser for one minute, using 16Hz frequency and 400mJ energy before sealer application.

Materials, instruments, equipment

Table 1 shows the materials, Instruments, and equipment used in the study.

**Table 1:** Materials, instruments, and equipment used in the study EDTA: ethylenediaminetetraacetic acid; Er:YAG: erbium-doped yttrium aluminium garnet laser; OCA: optical contact angle

Materials	Instruments	Equipment
Normal saline (Nirlife, Nirlife Healthcare Limited, Ahmedabad, Gujarat, India)	Ultrasonic scaler tip (Woodpecker, Guilin Woodpecker Medical Instrument Co. Ltd, Guilin, China)	Er:YAG laser (Fotona, Fotona d.o.o.,Ljubljana, Slovenia).
Glass jar with screw cap	Air rotor handpiece (NSK/Nakanishi inc., Kanuma Tochigi, Japan)	Contact angle analyzer (optical contact angle goniometer: OCA -15)
EDTA liquid 17% (Dentwash, Focus Dental, New Delhi, India;Prime Dent, Prime Dental Products Private Limited, Thane, Maharashtra, India)	Large round bur #BR 46 (Mani, Endo/Tech, Halifax, Canada)	
Bioceramic root canal sealer (BioRoot™ RCS, Septodont, Saint-Maur-des-Fossés, France).	Non-end cutting bur #EX 24 (Mani, Endo/Tech, Halifax, Canada)	
50 caries-free extracted single-rooted single canal permanent teeth	Endodontic explorer DG 16 (GDC Fine Crafted Dental Pvt. Ltd, Hoshiarpur, India)	
	Barbed broach (Dentsply Maillefer, Ballaigues, Switzerland)	
	Endodontic files #10 K, #15 K, #20 K – 25mm (Dentsply Maillefer, Ballaigues, Switzerland)	
	Double-sided carborundum disc (Kenda AG, Vaduz, Liechtenstein)	
	Straight handpiece (NSK/Nakanishi inc., Kanuma Tochigi, Japan)	
	Endobloc (Dentsply Maillefer, Ballaigues, Switzerland)	
	Indelible marker	
	Mixing pad	
	Spatula	
	Micropipette (Accupipet, Tarsons Products Limited, Kolkata, India)	
	Glass slide	

Statistical analysis

Data were entered and analyzed using IBM SPSS Statistics for Windows, Version 26.0 (Released 2019. IBM Corp., Armonk, New York, United States). Confidence intervals were set at 95%, and a p-value ≤ 0.05 was considered statistically significant. Descriptive statistics were used to summarize the wettability. One‑way ANOVA was performed and Post-hoc Tukey’s test was used for intergroup comparison.

## Results

Table 2 illustrates the comparison of CA values between five groups. CA of the control group is 53.14+2.44, which decreases in Er:YAG laser using 8Hz frequency and 200 mJ energy 43.15+1.73. In Er:YAG laser for one minute, using 8Hz frequency and 400mJ energy before sealer application, it was 41.69+1.50; in Er:YAG laser for one minute, using 16 Hz frequency and 200mJ energy before sealer application, it was 29.44+2.04. CA was lowest in Er:YAG laser for one minute, using 16Hz frequency and 400mJ energy before sealer application 19.98+3.26 (Table 2).

**Table 2:** Comparison of contact angle measurements in different groups Er:YAG: erbium-doped yttrium aluminum garnet

Bioceramic root canal sealer	Contact angle measurements	F, df
	Mean ± SD	
Control Group	53.14±2.44	637.9, 4
Er:YAG laser using 8Hz frequency and 200mJ energy	43.15±1.73	
Er:YAG laser using 8Hz frequency and 400mJ energy	41.69±1.50	
Er:YAG laser using 16Hz frequency and 200mJ energy	29.44±2.04	
Er:YAG laser using 16Hz frequency and 400mJ energy	19.98±3.26	

Table 3 illustrates the multiple comparisons of the mean difference in CA values between five groups. RC sealer adherence is primarily determined by the intraradicular dentin surface's relative surface free energy (wetting ability). Thereby, CA is a sizable measure demonstrating a liquid's wettability. The tendency of a liquid of spreading on a solid surface is measured by the establishment of CA. Liquid wets substrate when the CA is less than 90°; if it is larger than 90°, it's said to be non-wetting. Complete wetting is represented by a CA of zero. A better interaction connecting solid surfaces and liquids is often associated with low CAs. The liquid should have the smallest feasible CA with the surface for optimal wettability. The surface with a low CA or higher surface free energy has a higher wettability, which implies that the spreading and interaction of the sealer is better in a solid with high surface energy, resulting in a lower CA. Because the RC sealers employed in this investigation are liquids, their wettability on the RC dentin can be measured using the CA method. Our test findings demonstrated that the control group had the highest CA (low wettability) and it was statistically significant (p<0.05) with all other groups. There was no significant difference (p<0.05) in the wettability linking Er:YAG laser using 8Hz frequency and 200mJ energy and Er:YAG laser using 8Hz frequency and 400mJ energy. But there was a significant difference (p<0.05) between the other two groups. (Er:YAG laser using 16Hz frequency and 400mJ energy and Er:YAG laser using 16Hz frequency and 200mJ energy).

**Table 3:** Pairwise comparison of contact angle for different groups * : Statistically significant Er:YAG: erbium-doped yttrium aluminum garnet

Bioceramic root canal sealer	Control Group	Er:YAG laser using 8Hz frequency and 200mJ energy	Er:YAG laser using 8Hz frequency and 400mJ energy	Er:YAG laser using 16Hz frequency and 200mJ energy	Er:YAG laser using 16Hz frequency and 400mJ energy
Control Group	-	0.001*	0.001*	0.001*	0.001*
Er:YAG laser using 8Hz frequency and 200mJ energy	0.001*	-	0.26	0.001*	0.001*
Er:YAG laser using 8Hz frequency and 400mJ energy	0.001*	0.26	-	0.001*	0.001*
Er:YAG laser using 16Hz frequency and 200mJ energy	0.001*	0.001*	0.001*	-	0.001*
Er:YAG laser using 16Hz frequency and 400mJ energy	0.001*	0.001*	0.001*	0.001*	-

## Discussion

The use of an RC sealer is to seal abnormalities of the RC wall such as apical ramifications and deltas, in addition to places where initial root filling material finds it difficult to access, is called obturation. It also serves as a glue connecting the RC walls and filling substance of the root [22-24]. Our study used BioRoot RC sealant, which is a novel calcium silicate-based RC sealant that comes in powder and liquid form and was created exclusively for RC filling. The powder is mostly tricalcium silicate, while the liquid is an aqueous solution of calcium chloride and excipients, according to the manufacturer. When smear layer (SL) was conserved in an in vitro study by Turker et al. [25], calcium silicate-based sealers retained more than epoxy resin-based sealers. Following SL removal, BioRoot RCS had higher retention than MTA Plus (Prevest DenPro Limited, Jammu, India) and AH26 (Dentsply Maillefer, Ballaigues, Switzerland). Following SL removal, however, BioRoot RCS had a lower penetration depth than AH26 and MTA Plus. When the SL removal takes place, the relatively high fluidity of AH26 contacted exposed dentine tubules, which may have caused this. Our study aimed to evaluate and compare bioceramic RC sealer (BioRoot RCS) wettability on dentin with and without laser irradiation at frequencies of 8Hz and 16Hz and energies of 200 and 400mJ.

Irrigation solutions can modify dentin surface qualities including wetting ability, which can affect bacteria adhesion and govern the interface between dentin and restorative materials [14]. Furthermore, wettability is critical in determining an irrigant's appropriate contact time with dentinal walls. This feature is influenced by tubule density and is highly related to the chemical composition, roughness, and hydration state of dentin [26]. Wettability is linked to surface tension on ideal surfaces (chemically homogeneous, flat, nonreactive, undeformable, and not swelled by the wetting liquid) and, as a result, to dentin surface qualities [18].

The effect of these lasers on RC and surrounding dentine has been examined by several authors. The Er:YAG laser emits a suited wavelength at 2,940 nm, works through photoablation because its wavelength is quite close to the absorption of hydroxyapatite to the maximum. The water in tooth hard tissue is irradiated, evaporating instantly, ablating surrounding tissue via only minor thermal side effects. Hibst and Keller have demonstrated this in a number of studies [27]. Previously, the Er:YAG laser's applicability has been restricted to rigid delivery systems in no contact mode. This discovery about its improved light-conductive nature has expanded possibilities for this laser. Teeth with narrow or twisted RCs could also be managed without difficulty. Following all types of mechanical RC preparation, Er:YAG laser may remove diseased dentinal surfaces and the ubiquitous smear layer. The dentinal tubule orifices were exposed, allowing for a tight RC filling, that is required for success in endodontic therapy. Perin et al. [28] found that both the Er:YAG laser and 1% NaOCl proved successful against all microorganisms investigated up to the working length. The disinfection impact regarding erbium, chromium-doped yttrium, scandium, gallium, and garnet (Er,Cr:YSGG) laser in dentin is depending on output power, according to Schoop and his colleagues [29]. Gordon et al. [30] investigated the dentine disinfecting ability of this laser and found that as the laser duration or intensity rose, bacterial load decreased. The use of a two-minute laser showed better disinfection compared to the use of NaOCl. Post-treatment using the Er,Cr:YSGG and the neodymium-doped yttrium aluminum garnet (Nd:YAG) lasers at 1.5W, there was a reduction of 96% and 98%, respectively.

The free surface energies discharged in the control and acid-etch groups in our study were higher than those described in the literature [31,32]. These discrepancies could have occurred from a variety of experimental factors, such as the length of etching, the varied reference liquids utilized, the period between processing, and CA measurements. The energy disparities between enamel and dentin could have arisen from mineral and organic composition discrepancies. Hydroxyapatite contains a lot of energy and is quite reactive. SL removal is dependent on the fibreoptic tip and dentinal surface’s angle of incidence, in line with the findings of Brugnera et al. [33]. Lin et al. in 2001 [34] detected craters in human dentin treated using Nd:YAG laser using scanning electron microscopy (SEM), but Turkmen et al. in 2000 [35] found the dentinal surface to be uneven following irradiation with this laser. Fusion and enhanced surface imperfections are two of these properties that may provide an explanation for the same adhesion values obtained with the lasers as their frequency was raised.

Our study's use of human teeth as specimens was both a strength and a serious flaw. This study is meant to be translational, guiding clinicians' treatment of human patients. The benefits of using samples that are morphologically and anatomically representative of teeth seen in clinical practice are clear. However, the pool of specimens must have been relatively heterogeneous in terms of age, as this is known to affect the diameter of dentinal tubules, which could have contributed to tubule penetration variability. The sample size was less and so was a limiting factor. More extensive research on laser frequencies and energies is required for a study of this caliber. CA calculation can be done in a variety of ways.

## Conclusions

With the above findings, it can be concluded that the CA was lowest in Er:YAG laser for one minute, using 16Hz frequency and 400mJ energy before sealer application. The test findings demonstrated the control group to have the highest CA (low wettability) and it was statistically significant with all other groups. There was no significant difference in wettability between Er:YAG laser using 8Hz frequency and 200mJ energy and Er:YAG laser using 8Hz frequency and 400mJ energy. However, there was significant difference between the other two groups of Er:YAG laser using 16Hz frequency and 400mJ energy and Er:YAG laser using 16Hz frequency and 200mJ energy. Hence, lasers can be used for irradiation during RC treatment, and further in vivo trials can be carried out in the future.
